# Puerarin attenuates locomotor and cognitive deficits as well as hippocampal neuronal injury through the PI3K/Akt1/GSK-3β signaling pathway in an *in vivo* model of cerebral ischemia

**DOI:** 10.18632/oncotarget.22290

**Published:** 2017-11-07

**Authors:** Jinhao Tao, Yuehua Cui, Yu Duan, Nan Zhang, Congmin Wang, Fayong Zhang

**Affiliations:** ^1^ Pediatric Emergency and Critical Care Center, Children’s Hospital of Fudan University, Shanghai, P.R. China; ^2^ Department of Neurosurgery, Huashan Hospital Affiliated to Fudan University, Shanghai, P.R. China; ^3^ Department of Neurosurgery, Huadong Hospital Affiliated to Fudan University, Shanghai, P.R. China; ^4^ Department of Basic Medical Sciences, University of Arizona, Tucson, AZ, USA; ^5^ Department of Neurology, Affiliated Hospital of Hebei University of Engineering, Handan, P.R. China

**Keywords:** cerebral ischemia/reperfusion, puerarin, hippocampus, Akt, GSK-3β

## Abstract

Ischemic stroke causes irreversible damage to the brain. The hippocampus is a vulnerable region and plays an important role in cognition and locomotor activity. Puerarin is a phytoestrogen that has beneficial effects in treating neurological disorders. How puerarin protects against hippocampal injury and its molecular mechanisms remain to be elucidated. Transient global brain ischemia was induced by 4-vessel occlusion in adult male Sprague-Dawley rats. The rats were pretreated with puerarin alone or together with LY294002 (an PI3K inhibitor) before ischemia/reperfusion (I/R). The open- and closed-field tasks and Morris water maze (MWM) test were used to assess the effects of puerarin on anxiety-like behavioral and cognitive impairment following I/R. Hematoxylin-eosin staining(HE) was used to examine the survival of hippocampal CA1 pyramidal neurons, and immunoblotting was performed to examine the expression of the related proteins. By using the rat model for transient I/R, we demonstrated that puerarin pretreatment significantly increased the travelling distance and number of crossings in the open- and closed-field tests, reduced latency and increased the proportion of distance and time in zone IV in the MWM. The number of live cells in the hippocampus is sharply increased by puerarin pretreatment.We further observed that the levels of phosphorylated Akt1, GSK-3β and MCL-1were elevated and those of cleaved-caspase-3 were reduced in the puerarin-treatment group. Notably, the PI3K inhibitor LY294002 counteracted all of the effects of puerarin. Our findings suggest that puerarin protects the hippocampus from I/R damage by activating the PI3K/Akt1/GSK-3β/MCL-1 signaling pathway.

## INTRODUCTION

It has been estimated that millions of people suffer from ischemic stroke annually [1]. Ischemic stroke, caused by the rupture or occlusion of blood vessels, can compromise cell function and disrupt the equilibrium of brain structures, thus leading to complex brain disorders [2, 3]. One of the vulnerable regions in the brain is the hippocampus that plays important roles in spatial navigation, memory and locomotor activity [4, 5]. People with hippocampal damage may lose the ability to form and retain memories. Investigations regarding hippocampal protection offer a new angle for stroke therapy.

Puerarin is a phytoestrogen extracted from Pueraria plants. Its pharmacological activities have been extensively investigated [6]. Various diseases, such as cardiovascular dysfunction, neurological disorder and liver injury, may be treated using puerarin [6]. For example, in the mouse model of Alzheimer’s disease, puerarin treatment attenuated learning-memory deficits and inhibited apoptosis by upregulating Bcl-2 expression [7]. Puerarin has been demonstrated to display neuroprotective effects by suppressing apoptotic cell death in Parkinson’s disease models [8]. It has also been suggested that puerarin significantly reduces infarct size in transient middle cerebral artery occlusion (MCAO) models [9]. However, it is still unknown whether puerarin protects the hippocampus from global ischemia/reperfusion (I/R) damage.

The molecular mechanisms underlying puerarin’s activities are complex. Several signaling pathways have been suggested to be involved, such as, PI3K/Akt, CaMKII/AMPK, NO/NO-cGMP, JAK2/STAT3, TNFR1/FADD/caspase, cAMP/PKA, and AMPK/ mTOR [6]. Among these, the PI3K/Akt pathway plays important roles in cell survival and growth [10]. A variety of stimulations, such as stress and phosphatase inhibitors, can activate phosphatidylinositol 3-kinase (PI3K). The serine/threonine kinase, Akt, can be phosphorylated at definite sites and activated by PI3K [11]. Akt has 3 isoforms–Akt1, Akt2, Akt3–in mammalian species, and the first two are widely expressed in the brain [12, 13]. Active Akt can phosphorylate and activate/inhibit certain molecules, leading to glycolysis and/or anti-apoptotic enhancement [14]. One of the direct substrates of Akt is glycogen synthase kinase 3β (GSK-3β). This kinase is toxic for neurons, but its phosphorylated form has pro-survival functions [15]. Phosphorylation of GSK-3β deactivates its catalytic activity, causing the accumulation of myeloid cell leukemia-1 (MCL-1) protein [15, 16]. MCL-1 is an anti-apoptotic protein belonging to the bcl-2 family and is often over-expressed in various cancers, such as acute myelogenous leukemia and hepatocellular carcinoma [16, 17]. It has been demonstrated that puerarin prevents PC12 cells from undergoing apoptosis through the PI3K/Akt signaling pathway [18, 19]. Based on these findings, we wanted to find out whether puerarin promotes hippocampal cell survival through the PI3K/Akt/GSK-3β/MCL-1 cascade in the I/R rat model.

In this study, we used a rat model for transient global I/R to demonstrate that puerarin pretreatment significantly improves hippocampus-related behaviors and evidently promotes hippocampal cell survival. Through western blotting, we revealed that the phosphorylation levels of Akt1 and GSK-3β and the protein levels of MCL-1 are elevated. While total caspase-3 expression does not change, cleaved-caspase-3 is reduced in the puerarin-addition group. LY294002 (an PI3K inhibitor) counteracted all the effects of puerarin. These results indicate that puerarin benefits the hippocampus through the PI3K/Akt1/GSK-3β/MCL-1 signaling cascade in the I/R rat model. This study provides a basis for the clinical utilization of puerarin in ischemia-induced brain diseases.

## RESULTS

### Puerarin attenuated cognitive deficits and locomotor activity impairment

**Figure 1:**
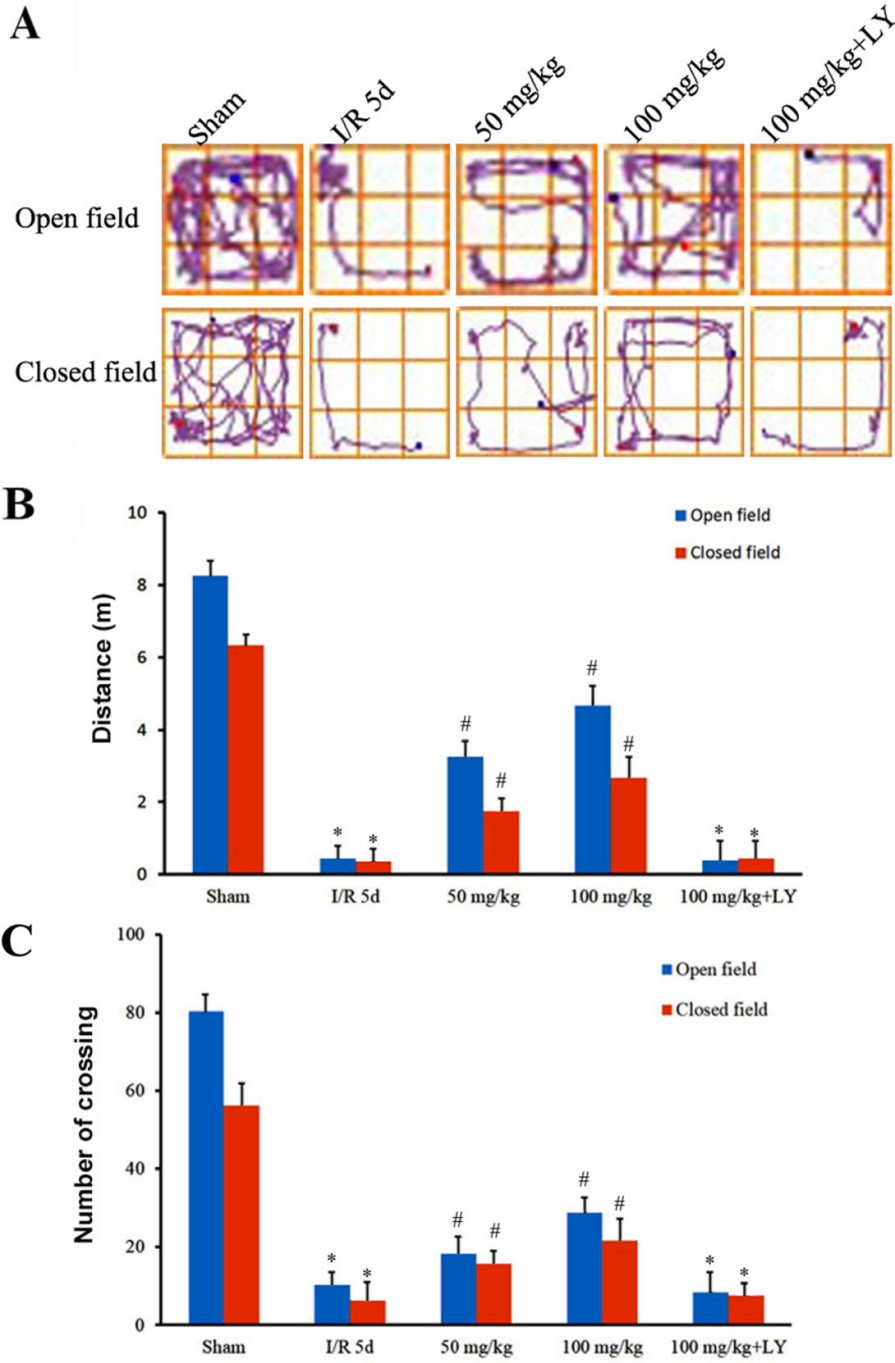
Puerarin attenuated locomotor activity impairment after I/R (**A**) The track maps of different groups. (**B**) Total distance traveled during 5 min. (**C**) Crossing lines during 5 min. Data are presented as the mean ± SD and analyzed by one-way ANOVA followed by the Newman–Keuls test. *p < 0.05, relative to the sham group. ^#^*p* < 0.05, relative to the I/R group.

To investigate whether puerarin improves spatial learning and memory as well as locomotor activity damaged by ischemia/reperfusion (I/R), we performed open- and closed-field tests and Morris water maze experiments. Figure 1 demonstrates that the distance travelled by I/R rats in an open-field was severely reduced, and puerarin pretreatment significantly increased that distance. Changes in the distance travelled in a closed-field were consistent with those travelled in the open-field (Figure 1). In addition, while I/R rats demonstrated sharply decreased crossing in both open- and closed-field, the puerarin pretreatment groups demonstrated significantly more crossing in both fields but their levels did not return to those observed in the sham group (Figure 1).

**Figure 2:**
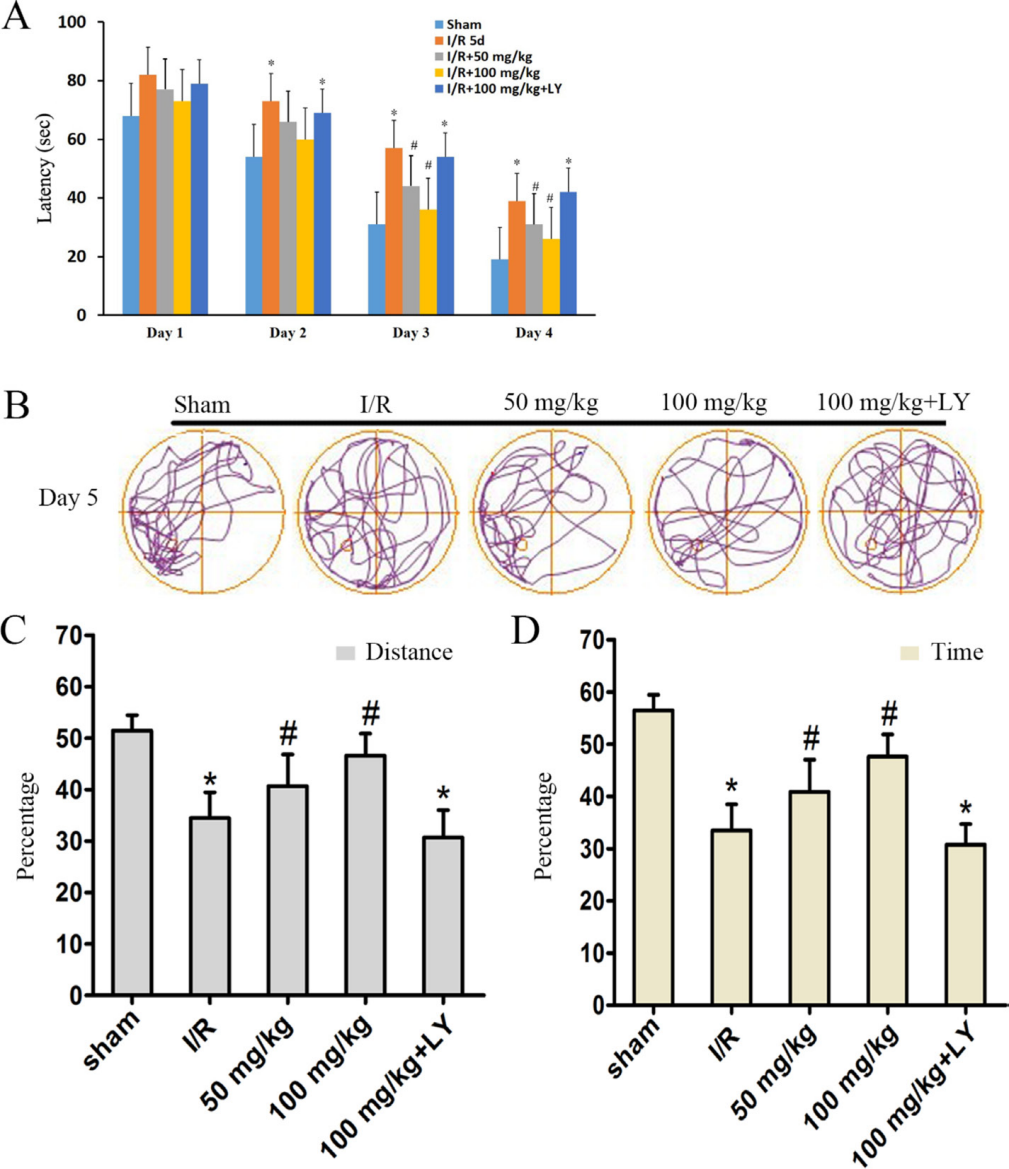
Puerarin ameliorated memory deficits in I/R rats On each day of training, the escape latency (**A**) of each group was measured. (**B**) Computer printouts of the swimming trajectories of each group on day 5. The circle represents the platform location. (**C** and **D**) The ratio of distance and time spent in the targeted quadrant when the platform was taken away. (A) Data are presented as the mean ± SD and analyzed by one-way ANOVA followed by the Newman–Keuls test. (C) and (D) data are presented as P50, *p < 0.05, relative to the sham group. ^#^*p* < 0.05, relative to the I/R group.

In the Morris water maze test, all rats were trained for 4 days. The latency of I/R rats was significantly higher than that of the sham rats by day 2 (Figure 2A). In the puerarin-pretreatment groups, the latency in the first two days was not significantly different from that of the I/R group, however, latency was significantly reduced by day 3 compared to that observed in the I/R group (Figure 2A). On the 5^th^ day, we measured spatial memory in the 5 groups. In this test, we removed the platform, and recorded the distance covered and the time spent by rats in the targeted quadrant of the Morris water maze (Figure 2B). The ratio of distance and time illustrated that the sham group had a preference for zone IV where the platform was located previously, whereas the I/R group did not exhibit this preference (Figure 2B–2D). Puerarin increased the proportion of distance and time spent in zone IV (Figure 2B–2D). These data conclusively suggested that puerarin attenuated the effects of ischemia, resulting in the amelioration of deficits in locomotor activity and cognitive behavior.

**Figure 3:**
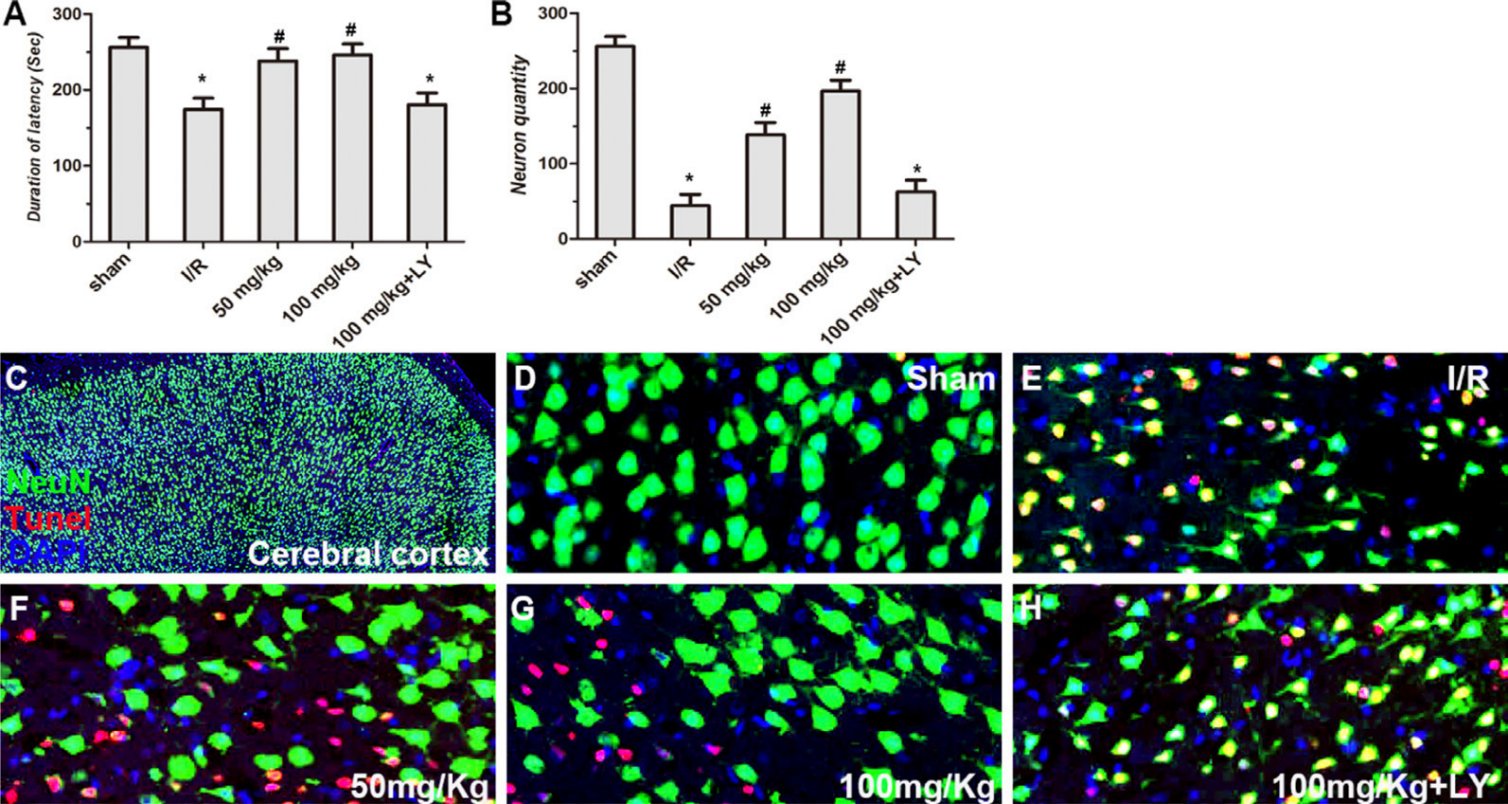
Puerarin improved cortical cell survival in I/R rats (**A**) Performance of rats in the rota-rod test after I/R and treatment of puerarin. Latency time (sec) of each group was measured. (**B**) The density of NeuN+/TUNEL- neurons (cells/mm^2^) in the cortex was quantitatively analyzed in each group. (**C**–**H**) NeuN staining, TUNEL assay and DAPI staining in the cortex in each group. Values are expressed as the mean ± SD (*n =* 7) and analyzed by one-way ANOVA followed by the Newman–Keuls test. *P < 0.05, relative to control group. ^#^*P* < 0.05, relative to I/R group.

Compared to the sham group, walking time wasseverely reduced in I/R rats in the rota-rod test, while puerarin at doses of 50 and 100 mg/kg significantly increased walking time after I/R. However, LY294002 blocked the actions of puerarin and the walking time was reduced to a level similar to that of the I/R group (Figure 3A).

### Puerarin improved cortical and hippocampal cell survival

**Figure 4:**
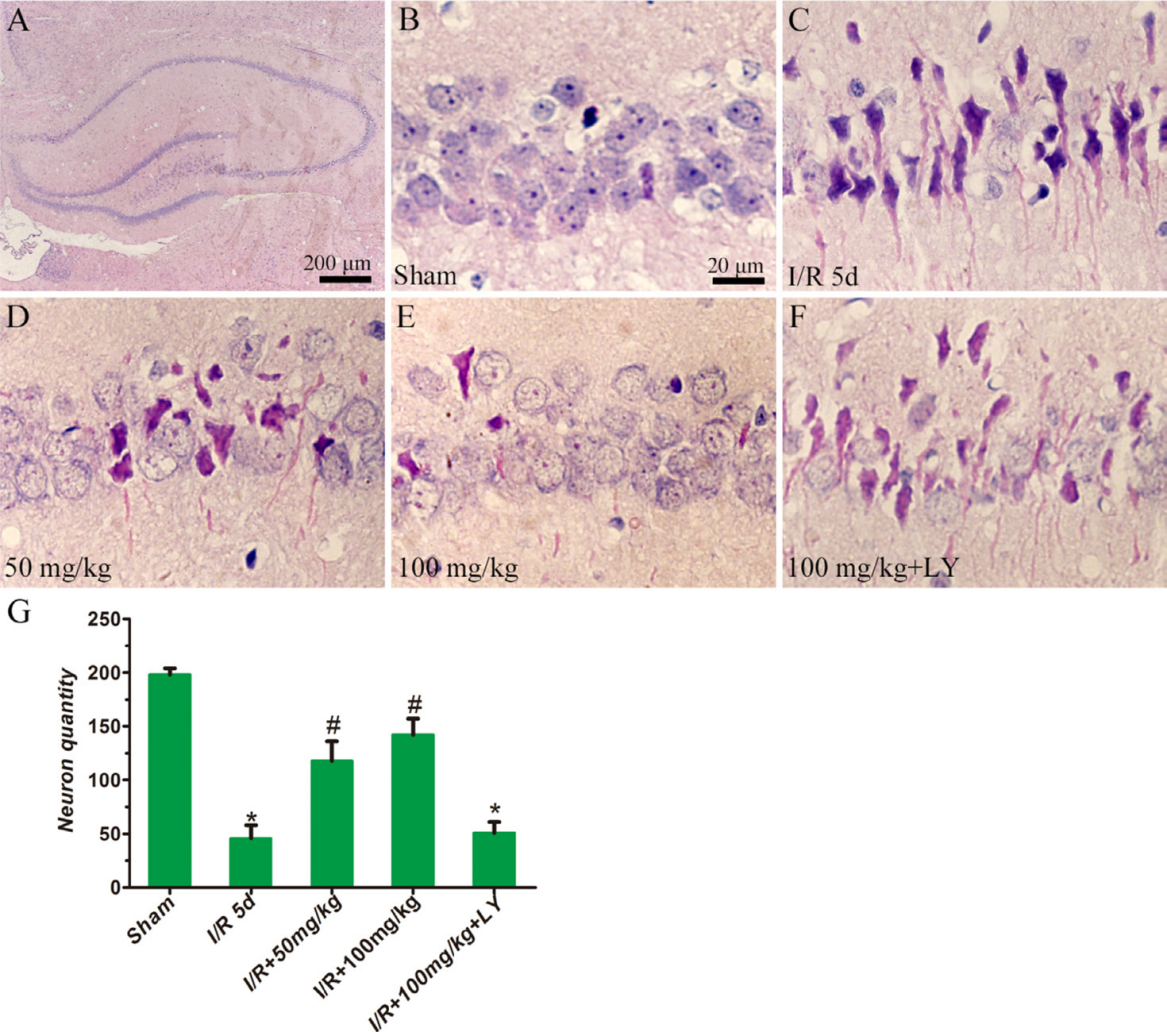
Puerarin inhibited hippocampal cell death at 5 days in I/R rats HE staining was performed on sections of rat brains. (**A**–**F**) Representative hippocampal photomicrographs of HE staining. (**G**) The density of cresyl violet-negative neurons (cells/mm^2^) in the hippocampus was quantitatively analyzed. Data are presented as the mean ± S D and analyzed by one-way ANOVA followed by the Newman–Keuls test. *p < 0.05, relative to the sham group. ^#^*p* < 0.05, relative to the I/R group.

To investigate whether puerarin protects hippocampal neurons from death, TUNEL staining and HE staining were used to examine the number of apoptotic and dead cells (Figures 3 and 4). In the cortex, compared to the sham group, the I/R group demonstrated extensively less neuronal survival (NeuN+/Tunel- cells), while pretreatment with puerarin demonstrated a neuroprotective function against I/R damage (Figure 3B–3H). In the hippocampus, compared to the sham group, a greater number of dead cells with irregular morphology and deeper staining were observed in the I/R group (Figure 4A–4C, 4G). The number of dead cells was significantly reduced in the puerarin-pretreatment group (Figure 4D–4E, 4G). Thus, these results suggest that puerarin may protect cortical and hippocampal cells from I/R-induced death.

### Puerarin improved Akt1 phosphorylation

**Figure 5:**
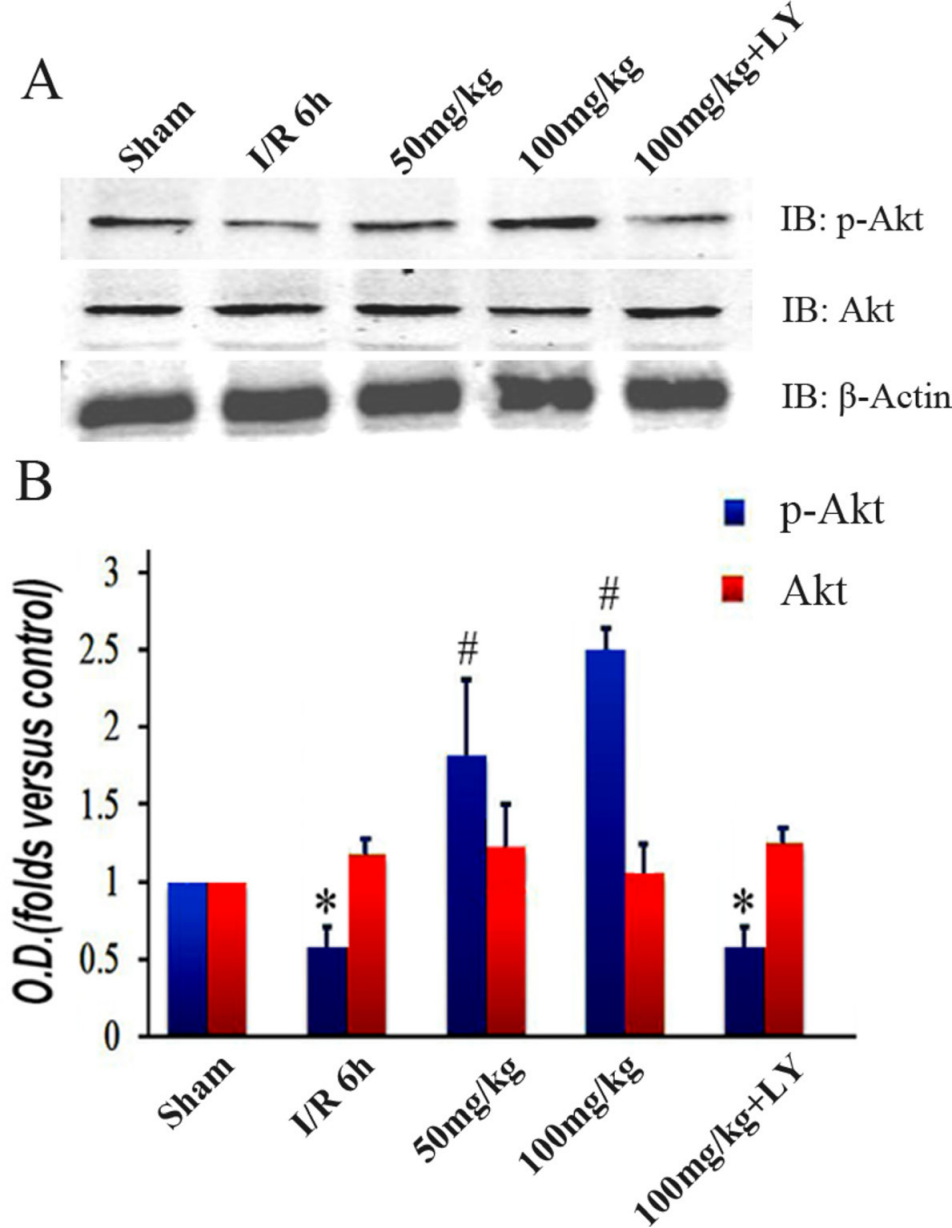
Puerarin increased phosphorylated Akt1 (Ser473) in the hippocampus after I/R-induced injury (**A**) Immunoblot bands were scanned. (**B**) The intensity of the bands was expressed by optical density (O.D.) analysis. Data are presented as the mean ± SD and analyzed by one-way ANOVA followed by the Newman–Keuls test. *p < 0.05, relative to the sham group. ^#^*p* < 0.05, relative to the I/R group.

While puerarin improved hippocampus-related behaviors and cell survival, the addition of LY294002 (an PI3K inhibitor) counteracted all its neuroprotective effects (Figures 1A–1B, 2A–2D, 3B–3H, and 4F–4G). This indicates that the PI3K signaling pathway may be important for these processes. To explore the involvement of this pathway, we examined PI3K’s target Akt1 and its phosphorylated form. While there was no difference in the total protein levels among the 5 groups, the expression of phosphorylated Akt1 (Ser473) was altered (Figure 5A and 5B). In contrast to the decreased level in the I/R group, puerarin pretreatment significantly enhanced Akt1 (Ser473) phosphorylation. Meanwhile, LY294002 blocked the actions of puerarin and phosphorylated Akt1 (Ser473) was reduced to a level similar to that observed in the I/R group (Figure 5A–5B).

### Puerarin led to increased GSK-3β phosphorylation and MCL-1 accumulation

**Figure 6:**
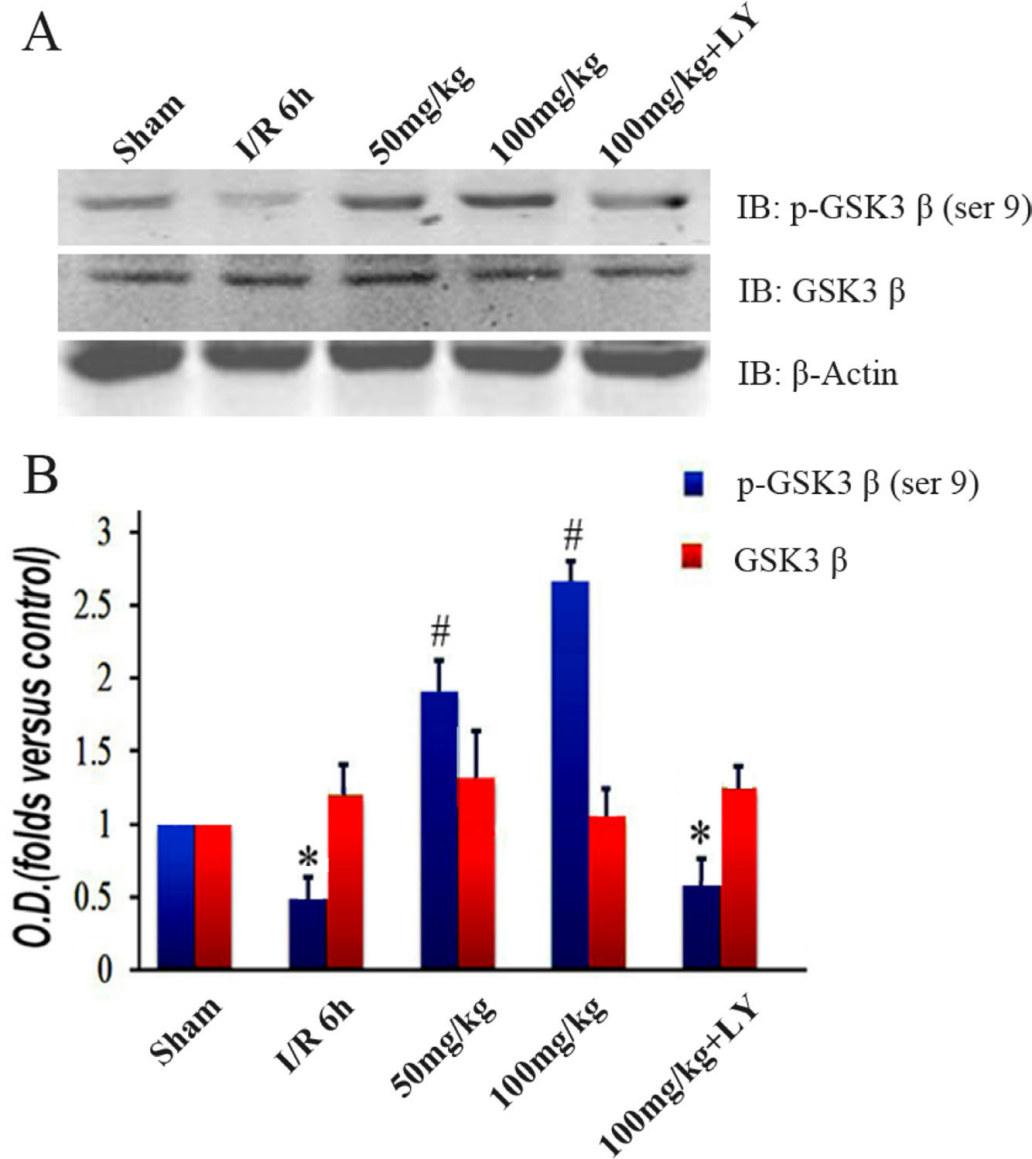
Puerarin increased phosphorylated GSK-3β (Ser9) in the hippocampus after I/R-induced injury (**A**) Immunoblot bands were scanned. (**B**) The intensity of the bands was expressed by optical density (O.D.) analysis. Data are presented as the mean ± SD and analyzed by one-way ANOVA followed by the Newman–Keuls test. *p < 0.05, relative to the sham group. ^#^*p* < 0.05, relative to the I/R group.

**Figure 7:**
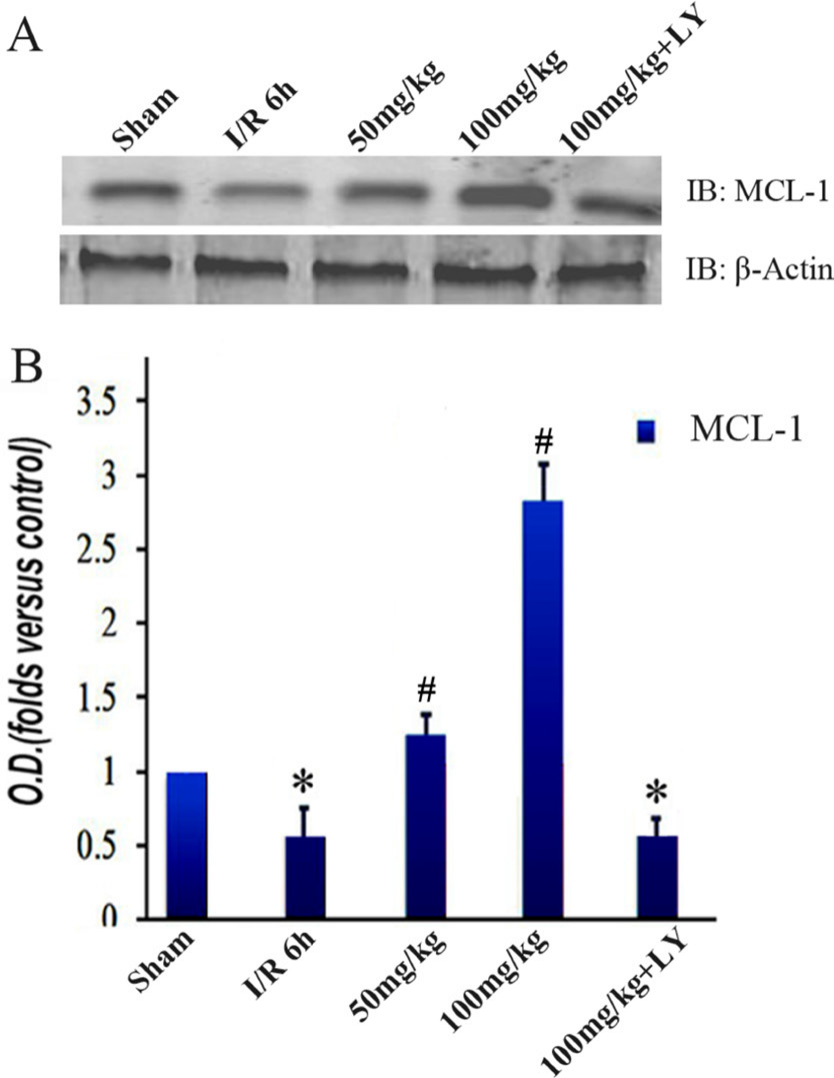
Puerarin increased MCL-1 in the hippocampus after I/R-induced injury (**A**) Immunoblot bands were scanned. (**B**) The intensity of the bands was expressed by optical density (O.D.) analysis. Data are presented as the mean ± SD and analyzed by one-way ANOVA followed by the Newman–Keuls test. *p < 0.05, relative to the sham group. ^#^*p* < 0.05, relative to the I/R group.

To investigate the downstream changes in the PI3K/Akt1 pathway, we detected the expression level of GSK-3β and its phosphorylated form. Similar to the Akt1 results, the total GSK-3β expression was consistent among the 5 groups. However, the levels of phosphorylated GSK-3β (Ser9) were lower in the I/R group than those in the sham group. These levels increased with puerarin pretreatment (Figure 6A and 6B). The extent of the improvement was in line with the puerarin concentration, suggesting a specific induction by puerarin (Figure 6B). To further probe downstream targets, we checked the protein levels of MCL-1. Consistent with the change in GSK-3β phosphorylation, MCL-1 was downregulated in the I/R group, but upregulated in the puerarin pretreatment groups (Figure 7A–7B). More drastic improvements appeared in the 100 mg/kg puerarin pretreatment group (Figure 7A–7B). LY294002 inhibited GSK-3β phosphorylation and thus reduced the levels of MCL-1.

### Puerarin blocked the elevation of cleaved-caspase-3 in the I/R group

**Figure 8:**
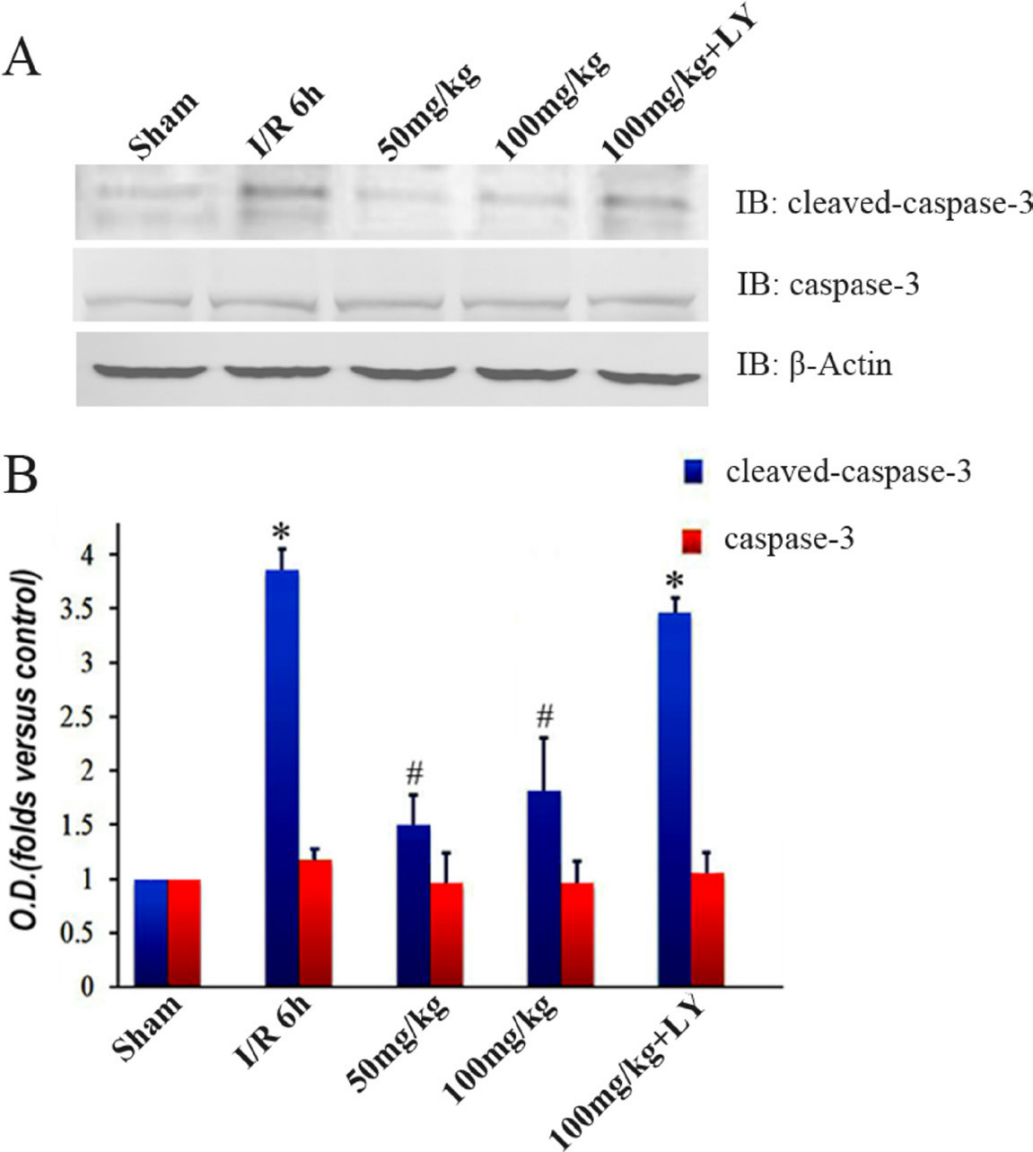
Puerarin inhibited cleaved caspase-3 in the hippocampus after I/R-induced injury (**A**) Immunoblot bands were scanned. (**B**) The intensity of the bands was expressed by optical density (O.D.) analysis. Data are presented as the mean ± SD and analyzed by one-way ANOVA followed by the Newman–Keuls test. *p < 0.05, relative to the sham group. ^#^*p* < 0.05, relative to the I/R group.

To explore the effects of puerarin on cell apoptosis, we detected the expression level of caspase-3. Levels of caspase-3 protein were not significantly different among the 5 groups (Figure 8A–8B). The protein detected by cleaved-caspase-3 antibody was drastically increased in the I/R group, but was inhibited by puerarin pretreatment (Figure 8A–8B). Suppression of cleaved-caspase-3 by puerarin was blocked by LY294002 (Figure 8A–8B).

## DISCUSSION

In this study, we demonstrated that puerarin ameliorates defects in locomotor activity and spatial-memory behaviors, and improves hippocampal neuronal survival in the brain with transient global cerebral ischemia. Upon treatment with puerarin, activity of the p-Akt1/p-GSK-3β/MCL-1 cascade is upregulated and activation of the apoptotic protein caspase-3 is blocked.

The hippocampus is important for cognition and memory formation. Mounting evidence suggests that ischemia causes severe neuronal damage in the hippocampus and leads to impairments of learning and memory [5]. Although the hippocampus is well known to be sensitive to ischemia, the vulnerability of its sub-regions differs: while the CA1 region is the most vulnerable, the other three regions are relatively more resistant to ischemia [20, 21]. Diverse cellular changes, such as DNA damage and oxidative stress, are responsible for reperfusion injury following ischemia [21]. The damage in the hippocampus is called “delayed neuronal death”. Hippocampal neurons degenerate after two to four days of reperfusion [20]. Several studies have focused on delayed neuronal death to develop preventative interventions for ischemic patients [20, 21]. Based on these studies, our data here described the effects of puerarin pretreatment up to 5 days after injury (Figures 1–8). We observed evident protective effects at this critical time point when I/R causes brain damage; thus, the speculation that neuroprotection provided by puerarin will be long-lasting is reasonable.

Puerarin–one of the active ingredients in herbal medicine–has protective functions in multiple tissues, such as bone, liver, the cardiovascular system and the brain [6]. Studies have demonstrated that in rats with cerebral I/R injury, puerarin enhanced antioxidant capacity, removed lipid peroxidation products, improved antioxidant activity, and reduced the degree of focal ischemic injury [22]. Here we demonstrated that puerarin-pretreatment improves behaviors in I/R rats, as observed the MWM test, thereby suggesting that puerarin benefits the recovery of hippocampus-related behaviors. Moreover, through cresyl violet staining, we observed that a greater number of cortical and hippocampal neurons survive in the puerarin administration group (Figures 3–4), indicating that puerarin protects cortical tissues from I/R damage.

The effects of puerarin in various conditions depend on what it is binding to. In a rat model with aconitine-induced arrhythmias, puerarin is a potassium channel blocker [23]. In isoprenaline-induced fibrotic myocardial tissue, puerarin activates the peroxisome proliferator-activated receptor [24]. In dorsal root ganglion neurons, puerarin acts on P2X_3_ receptors to alleviate neuropathic pain [25]. Puerarin also exhibits anti-osteoporosis effects through a non-estrogen receptor in ovariectomized mice [26]. Our western blot results revealed that the p-Akt1/p-GSK-3β/MCL-1 cascade is activated by puerarin during I/R (Figures 4–6), indicating the involvement of the PI3K/Akt1 signaling pathway. In ischemic cardiomyocytes, puerarin binds to an estrogen receptor to activate the PI3K/Akt-dependent signaling pathway [27]. We speculate that an estrogen receptor may be the binding site of puerarin in the I/R-affected hippocampus.

Ischemia/Reperfusion triggers intrinsic and extrinsic pathways of apoptosis [28]. Apoptosis contains three different signaling pathways: mitochondria-dependent, endoplasmic reticulum (ER)-dependent, and receptor-dependent pathways [29]. Among these, the mitochondria-dependent pathway is the dominant one in the I/R-affected brain [30]. Cytochrome c released from mitochondria leads to the autoactivation of procaspase-9 [31], resulting in the activation of caspase-3 and DNA fragmentation [32]. Caspase-3 is a crucial protein during apoptosis, and inhibition of its activation can protect cells from death [33]. *In vitro* data reveal that puerarin inhibits the activation of caspase-8 and caspase-3, leading to improvement in cell survival [34]. It has been reported that puerarin (100 μmol/L) prevents cultured hippocampal neurons from death *in vitro* [9]. The level of active Akt1 is strongly correlated with the amount of cell death during ischemic stroke [35]. By analyzing our data in the I/R model, we demonstrated that puerarin inhibits the accumulation of cleaved-caspase-3, but not caspase-3 (Figure 8), thereby suggesting that puerarin attenuates cell death by blocking the activation of caspase-3. Notably, the addition of LY294002 counteracts all the effects of puerarin, indicating that PI3K/Akt1 is the primary pathway mediating the protection of hippocampus from ischemic impairments in the puerarin-pretreatment group *in vivo*. The full activation of Akt needs the phosphorylation of both the Thr308 and the Ser473 sites. While the phosphorylation of Thr308 in the kinase domain partially activates Akt, phosphorylation of Ser473 in the C-terminal domain is critical for full activation [36].

In conclusion, this work is novel and provides new insights into the protective function and potential mechanisms of puerarin in an *in vivo* model of transient global cerebral ischemia and thus offers potential targets for therapeutic intervention. The severe damage caused by I/R to the hippocampus greatly affects the daily lives and health of patients. The increasing incidence of I/R needs improved efforts towards prevention and treatment explorations that correlate with the time and dosage of puerarin administration. It is worth studying whether applying puerarin after ischemic insult will favor clinical therapy. In this study, the detailed mechanisms underlying puerarin’s actions are not completely elucidated. How puerarin activates the PI3K/Akt signaling pathway awaits further address. It will be interesting to reveal the molecular mechanisms of puerarin in individual cells and distinguish its cell-autonomous and non-cell-autonomous roles. Investigation on these issues in the future will favor the clinical utilization of puerarin.

## MATERIALS AND METHODS

### Animals and antibodies

All animal experiments were done in accordance with the guidelines of Fudan University. Adult male Sprague–Dawley rats weighing 200–250 g (ShanghaiExperimental Animal Center, Chinese Academy of Science, Shanghai, China) were given free access to food and water before surgery.All experiments were performed on adult Sprague–Dawley rats housed at a constant temperature. Puerarin and LY294002 (Akt1 pathway inhibitor) were purchased from Sigma-Aldrich (St. Louis, USA). Antibodies against Akt, MCL-1 and β-actin were purchased from Santa Cruz Biotechnology. P-Akt (Ser473), GSK-3β, GSK-3β (Ser9), Caspase-3 and cleaved Caspase-3 antibodies were purchased from Cell Signaling Biotechnology. These antibodies have been validated in previous studies [15, 16, 37].

### Rat model of cerebral ischemia/reperfusion (I/R)

Transient global cerebral ischemia (15 min) was induced by four-vessel occlusion (4-VO), as described previously [38]. In our experiment, rats were randomly assigned to five groups: Sham, I/R, I/R + 50 mg/kg puerarin, I/R + 100 mg/kg puerarin, I/R + 100 mg/kg puerarin + LY (*n =* 15 in each group). In the I/R groups, we administered the rats with a general anesthetic, pentobarbital (350 mg/kg, intraperitoneal injection). Following anesthesia, we occluded the two vertebral arteries of rats permanently by electrocautery and exposed the common carotid arteries. Next, the rats were subjected to recovery and fasting overnight. To induce ischemia, we used aneurysm clips to occlude the common carotid arteries for 15 min. Thereafter, the clips were removed for reperfusion. Rats in the sham group were treated with the same surgical procedures without the occlusion of vessels. All efforts were made to minimize the suffering endured by rats and to reduce the number of rats used.

### Administration of drugs

The administration of puerarin used in this study was performed based on a previous study [39]. Puerarin (50 mg/kg or 100 mg/kg) was dissolved in 10% methyl glycol and injected intraperitoneally 2 h before bilateralcarotid artery occlusion. The kinetic analysis of puerarin revealed a half-life of 46.9 minutes [40]. LY294002 is a strong inhibitor of PI3Kthat is the major mode of activation of Akt; thus, it can efficiently inhibit PI3K/Akt activities and has been widely used in studies regarding the PI3K/Akt pathway [41, 42]. The puerarin +LY group was administered with puerarin (100 mg/kg) and LY294002 (10 mg/kg, dissolved in DMSO) intraperitoneally at the same time as the other groups.

### Open-field and closed-field tests

The open-field and closed-field tests were used to evaluate locomotive activity. Each rat was placed in the center of an open-field apparatus or a closed-field apparatus (W50 Χ D50 Χ H30 cm) and acclimated for 3 min. Next, their freely moving behavior was monitored for 5 min. Their behavior was analyzed using the ANY-maze Video Tracking System (Stoelting, Wood Dale, IL, USA) with a CCD camera; the total distance traveled and the number of crossings were analyzed.

### Rota-rod test

To evaluate motor coordination and balance, all rats were subjected to a rota-rod test. Rats were trained on a gradually accelerating (4–35 rpm) rota-rod (7750 Ugo Basile, Italy). Each animal was trained for a minimum of three trials, each lasting for 5 min, to allow the animal to learn the task and establish a baseline performance. On the experimental day, the time spent walking on the rota-rod without falling was measured twice per animal, as described in a previous study [43]. The interval between each trial was 15 min. The mean of the two trials was calculated for each rat.

### Morris water maze (MWM) test

As described previously, the Morris water maze test was used to evaluate spatial learning and memory [44]. Two sessions of four trials were conducted on the first testing day, with an interval of 4 h. The first session was considered as a training procedure. The second session was the formal test and was conducted daily for the next five days. Four hours after the last trial, a probe trial was performed for 90 s in which the platform was removed from the tank. The rat was placed in the water at the same random start location, and the time spent in the quadrant which previously contained the platform was recorded.

### Histology

In the 4-VO ischemic model, rats were anesthetized with chloral hydrate and underwent transcardial perfusion with 0.9% saline, followed by 4% paraformaldehyde in 0.1 M phosphate buffer (PBS). Brains were removed, post-fixed overnight in paraformaldehyde, processed, and embedded in paraffin. Coronal brain sections (6 μm thick) were cut on a microtome. Sections were deparaffinized in xylene and rehydrated in a gradient of ethanol and distilled water. Sections were stained with cresyl violet and examined using a light microscope; the neuronal density of the hippocampal CA1 pyramidal cells was expressed as the number of cells per 1 mm length counted under a light microscope (3400). Neuronal survival was quantitatively analyzed by counting the number of surviving neurons within 0.02 mm^2^.

### Cresyl violet staining

Embedded brains were cut on a microtome to obtain coronal brain sections (6 μm thick). The coronal sections were deparaffinized in xylene and then rehydrated in a gradient of ethanol and distilled water. Thereafter, the sections were stained with cresyl violet. Slides were examined under a light microscope, and the neuronal density of the hippocampal CA1 pyramidal neurons (cells/mm^2^) was calculated.

### TUNEL staining and immunostaining

The number of apoptotic cells was determined using an *In Situ* Cell Death Detection Kit (TMR Green; Roche Applied Sciences, Indianapolis, IN) that allows visualization of terminal deoxynucleotidyl transferase dUTP nick end labeling (TUNEL). TUNEL+ nuclei were visualized using confocal microscopy and were quantified using Image-J. As NeuN is a specific neuronal marker [45], we performed an immunohistochemistry assay to stain NeuN. A monoclonal rabbit anti-NeuN primary antiserum (1:1000) was used, followed by Alexa Fluor 488 goat-anti-rabbit secondary antiserum (1:1000). NeuN+ nuclei were visualized using the same confocal microscope as described for TUNEL staining.

### Western blotting

Western blotting was used to detect Akt and downstream proteins in the Akt signaling pathway. During cerebral ischemia, cell death primarily occurs through apoptosis. Caspase-3 is a cysteine protease and acts as a key mediator of apoptosis. Because cleaved-caspase-3 is the active enzyme formed after cleavage [46], its protein level can be considered as an index of apoptosis [47]. At 24 h following I/R injury, rats were intraperitoneally anesthetized and sacrificed by cervical dislocation to obtain the brain hippocampus region. The hippocampus was collected and lysed. The supernatant was used to check for the protein levels. The membranes were blocked with 5% nonfat milk in Trisbuffered saline (TBS) at 37°C. Rabbit anti-Akt polyclonal antibody (1:500), rabbit anti-p-Akt (Ser473) monoclonal antibody (1:500), rabbit antiactin monoclonal antibody (1:2000), goat anti- GSK-3β polyclonal antibody (1:500), goat anti-phospo-GSK-3β polyclonal antibody (1:500), rabbit polyclonal anti-caspase-3 antibody (1:1000) and rabbit anti-cleaved caspase-3 antibody (1:1000) were diluted in freshly prepared PBS containing 3% skim milk powder, and blots were incubated with the primary antibodies at 4°C. Antigen-antibody complexes were detected using an enhanced chemiluminescence kit and bands were analyzed using the ImageJ version 1.42q software.

### Statistical analyses

The data for escape latency were analyzed by repeated measures analysis of variance based on Mauchly’s test of sphericity. Comparisons of distance and time percentage were made using the Kruskal-Wallis test, and multiple independent samples were compared using the Nemenyi test. We compared the remaining data using the Kolmogorov-Smirnov test and the homogeneity of variance test. Data were expressed as the mean ± standard deviation (SD). Data from behavioral tests and western blotting were analyzed by a one-way analysis of variance (ANOVA). To explain the exact difference between means, the Bonferroni test was applied. *P* values < 0.05 were considered as significant.
